# Epigenetic m6A RNA Modification Reader YTHDF1 in Merkel Cell Carcinoma

**DOI:** 10.3390/cancers15164075

**Published:** 2023-08-12

**Authors:** Aayami Jaguri, Martin Steinhoff, Aamir Ahmad

**Affiliations:** 1 Weill Cornell Medicine-Qatar, Doha 24811, Qatar; aaj4005@qatar-med.cornell.edu (A.J.); msteinhoff@hamad.qa (M.S.); 2 Translational Research Institute, Academic Health System, Hamad Medical Corporation, Doha 3050, Qatar; 3 Dermatology Institute, Academic Health System, Hamad Medical Corporation, Doha 3050, Qatar; 4 Department of Dermatology and Venereology, Rumailah Hospital, Hamad Medical Corporation, Doha 3050, Qatar; 5 Department of Dermatology, Weill Cornell Medicine, New York, NY 10065, USA; 6 School of Medicine, Qatar University, Doha 2713, Qatar; 7 School of Life Sciences, Hamad-Bin-Khalifa University, Doha 34110, Qatar

Merkel cell carcinoma (MCC) is a rare and aggressive neuroendocrine cutaneous malignancy that commonly affects older individuals with a high mortality rate [1]. The treatment of MCC is still a challenge. The presence of Merkel cell polyomavirus (MCPyV or MCV) is a major causative factor for the disease, associated with approximately 80% of MCC cases [2]. The only known polyomavirus virus to play a role in tumorigenesis, MCPyV, is a double-stranded DNA virus from the Polyomaviridae family of viruses and is known to target dermal fibroblasts. It induces oncogenesis by clonally integrating into the MCC tumor cell genome and expressing its oncogenic T antigens: large T (lT), small T (sT), and 57 kT antigens [3].

While the N6-methyladenosine (m6A) modification has not been documented in MCPyV mRNA, m6A residues are a known feature of the mRNAs of a closely related animal tumor virus, simian virus 40 (SV40), as well as numerous other viruses exhibiting nuclear replication [4,5]. In eukaryotes, m6A modifications are among the most abundant mRNA modifications and have been linked to several cancers, including breast, lung, and brain cancers [6,7]. m6A modifications play an important role in mRNA structure and function, facilitating the regulation of mRNA at different levels, including during splicing, processing, stability, and translation [8,9]. The m6A modifications present in the 3′ untranslated region (3′ UTR) of mRNA can be recognized in the mRNA transcript using the epigenetic reader YTHDF1, which binds to the mRNA and induces translation.

The dysregulation of the various components involved in m6A modification has been exhibited in several cancers, for instance, in the role of FTA in leukemia; however, no such regulatory mechanisms for m6A modification have been found for MCC as of yet [10]. In their article entitled “Oncogenic Role of an Epigenetic Reader of m6A RNA Modification: YTHDF1 in Merkel Cell Carcinoma”, published in the 12th volume of *Cancers*, 2020, Orouji et al. addressed the regulatory role of YTHDF1 in the tumorigenesis of MCC, utilizing the genomic-wide analysis of seven MCC cell lines (n = 4 MCPyV positive, n = 3 MCPyV negative) [11].

Their experimental strategy included copy number analysis, which was performed using a panel of ~300 k genome-wide tag single-nucleotide polymorphisms (SNPs). Transcriptomic analysis was then performed using transcription data from different cell lines. Based on copy number and transcriptomic analyses, two genes on chromosome 20—YTHDF1 and KCNQ2—were identified and amplified. Since the expression of YTHDF1 was found to be more consistent among the tested cell lines than that of KCNQ2, YTHDF1 was studied further. To evaluate whether this finding was consistent at the level of protein expression, the levels of YTHDF1 proteins and its major paralog YTHDF2 were evaluated through immunoblots with lysates from MCC and melanoma cell lines. All MCC cell lines were found to exhibit higher expression of YTHDF1 and YTHDF2 proteins than the melanoma cell lines.

The analysis was further focused on the immunohistochemistry of YTHDF1, which was performed on tissue microarrays from 31 MCC patients and 26 melanoma patients as the control cohort, revealing the YTHDF1 protein to be highly expressed in 74.2% of patient samples from MCC patients compared with only 3.8% of the samples from melanoma patients. To identify potential m6A sites in the clonally integrated sequence of the MCPyV genome of the WaGa cell line, a sequence-based RNA adenosine methylation site predictor (SRAMP) tool was used and a prediction score was determined. m6A modification was found to be present at two sites in the genomic sequence of MCPyV in the host cell. The role of YTHDF proteins was further explored through shRNA-mediated knockdown. Using two shRNAs, YTHDF1 and YTHDF2 were silenced in two MCC cell lines (WaGa and PeTa). Upon YTHDF1 depletion, the translational initiation factors eIF3A and eI3FB were found to be significantly downregulated. Finally, cell proliferation assays and colony formation assays were performed in MCC cell lines, revealing that the YTHDF1-silenced samples were less proliferative and showed less clonogenic potential compared with controls.

To summarize, Orouji et al. [11] present a novel mechanism for the tumorigenesis of MCC involving the upregulation and elevated expression of the m6A reader, YTHDF1, and the induction of translational initiation factors, eIF3A and 3B. While prior studies have noted the role of the small T protein in the modulation of cap-dependent translation, the results presented in this study indicate that the presence of m6A at multiple sites on the MCPyV genomic sequence, as well as the high expression of YTHDF1 proteins in MCC, also serve to enhance cap-dependent translation [3]. Furthermore, the study highlights the association between a high expression level of YTHDF1 and poorer prognosis and survival of MCCC patients. Targeting YTHDF1 to restrict the activation of translation could therefore be a potential therapeutic approach for the treatment of MCC. Another alternative strategy could be the use of clustered regularly interspaced short palindromic repeats (CRISPR-Cas) to edit the m6A from its site on the integrated MCPyV sequence, thus inhibiting the expression of YTHDF1 and preventing tumorigenesis.

This study is the first to elucidate the role of YTHDF1 in m6A identification in MCC and introduce a novel mechanism for the tumorigenesis of MCC involving the activation of cap-dependent translation. To further understand and validate the function of YTHDF1 in the tumorigenesis of MCC, future studies are needed to validate these findings. The study by Orouji et al. [11] notes that in vivo experiments such as those using YTHDF1 mouse models, clinical survival studies with a larger patient cohort, and the genome-wide mapping of m6A may help encourage further progress toward understanding the role of YTHDF1 and possibly improving treatment strategies for MCC in the future.
